# Analysis of femoroacetabular impingement by a triade of label-free optical spectroscopy techniques

**DOI:** 10.1038/s42003-025-08400-5

**Published:** 2025-07-02

**Authors:** Martin Hohmann, Lucas Kreiss, Faramarz Dehghani, Dongqin Ni, Max Gmelch, Oliver Friedrich, Lorenz Büchler, Michael Schmidt

**Affiliations:** 1 Institute of Photonic Technologies (LPT), Friedrich-Alexander-Universität Erlangen-Nürnberg (FAU), Erlangen, Germany; 2 Erlangen Graduate School in Advanced Optical Technologies (SAOT), Erlangen, Germany; 3 Institute of Medical Biotechnology (MBT), Friedrich-Alexander-Universität Erlangen-Nürnberg (FAU), Erlangen, Germany; 4 Institut für Anatomie und Zellbiologie, Martin Luther Universität Halle-Wittenberg, Halle (Saale), Germany; 5 Department for Orthopaedic surgery and Traumatology, Aarau, Switzerland; 6 Department of Orthopaedic Surgery and Traumatology, Inselspital, Bern Univeristy Hospital, University of Bern, Bern, Switzerland

**Keywords:** Osteoarthritis, Molecular medicine, Biochemical reaction networks, Data processing

## Abstract

Understanding the biochemical mechanisms of hip joint diseases remains challenging. Femoroacetabular impingement (FAI) is a condition where developmental deformities of the hip joint reduce mobility and cause tissue damage, with characteristic red staining observed in affected cartilage. Here we show how combining three complementary optical analysis techniques- laser-induced breakdown spectroscopy, Raman spectroscopy, and diffuse reflectance spectroscopy- reveals tissue alterations without invasive procedures. Our analysis identifies elevated amide I levels indicating bone damage, reduced hydroxypyroline showing active bone regeneration, and increased mineral components (phosphate and carbonate) reflecting bone hardening. These bone healing processes elevate tyrosine levels in the affected tissue. When exposed to air, this excess tyrosine rapidly oxidizes through enzymatic pathways to form pheomelanin, producing the distinctive red staining. This biochemical model explains both the compositional changes and visual characteristics of FAI-affected tissue, demonstrating how non-invasive optical techniques can elucidate complex disease mechanisms in joint pathologies.

## Abstract (web summary)

FAI hip joint deformities cause red-stained cartilage. Using LIBS, Raman, and reflectance spectroscopy, we found bone damage/healing processes produce excess tyrosine. Upon air exposure, tyrosine oxidizes to pheomelanin, causing red staining.

## Introduction

Many biomedical investigations require the integration of multiple analytical approaches to provide comprehensive insights into complex biological processes. These technologies should be user-friendly and suitable for potential in vivo applications. Additionally, high spatial resolution is often crucial for detailed tissue analysis. The combination of Laser-induced breakdown spectroscopy (LIBS), Raman spectroscopy (RS), and diffuse reflectance spectroscopy (DRS) enables comprehensive tissue analysis through complementary measurements. These optical techniques share the benefits of being label-free, minimally invasive, non-destructive and contact-free. Moreover, this analytical approach eliminates the need for biochemical labels or tissue fixation procedures.

In LIBS measurements, a high-power laser pulse ablates a microscopic portion of the sample and a plasma is generated from the material under investigation. The recombination of elements within the plasma then generates emission of light that can be collected and analyszed by a spectrometer to measure the atomic and molecular emission lines^1^. Research on LIBS, particularly its capability to measure elemental composition, has gained increasing attention over recent decades^2^ especially since it allows measuring elemental concentrations in solids, liquids and gases^2^. The technique enables absolute quantification with precision at the parts-per-million level^3^. Recently, LIBS has shown promising results in medical applications^4^, for instance as potential feedback mechanism during surgery, i.e., to differentiate nerve and gland tissue^5^ or cartilage and bone^6^ or to trace elements and contaminants in tissues and fluids^7^ or metals and metalloids in teeth, hair, nails or bone^4^. LIBS has been combined with other modalities in the past, most commonly with RS and with Laser-induced Fluorescence Spectroscopy^8^, but also with newer technologies, like random lasers^9^.

RS, on the other hand, targets the vibrational and rotational energy bonds of molecules in the sample. It is a widely used method in many fields^10,11^, such as the assessment of food quality^12^, the analysis of carotenoids in biological matrices^10^, or in cancer research^13–15^. Normally, the Raman spectra of organic molecules are analysed by specific peaks^11^. For example, studies have shown that the ratio of amide I to amide III in human bone significantly increases with age^16^. RS is widely used in cell- and micro-biology research^17^, for tissue analysis^18^ or cancer research^19^. For instance, its high bio-chemical specificity was essential to reveal the biochemical pathway of an experimental anti-cancer treatment^20^. RS is often combined with other technologies, like LIBS and LIF^8^ multiphoton microscopy^21^, or random lasers^22^.

Femoroacetabular impingement (FAI) is characterized by reduced hip mobility due to early bone contact between the femur and acetabulum during movement. In many cases, the cause of the reduced range of motion is a deformity of the hip joint with an increased size of the anterior femoral neck (Cam-type FAI), a deep acetabulum (Pincer-type FAI), or a combination of both. Symptomatic Cam-type FAI is a relevant risk factor for the later development of hip osteoarthritis^23–28^. State of the art treatment for FAI is to restore normal hip anatomy to regain normal range of motion of the hip and the repair of damages to the acetabular labrum or cartilage to eliminate symptoms and prevent progression of osteoarthritis. In open treatment of FAI, the femoral head is dislocated from the acetabulum to gain access to the entire joint. After dislocation, the femoral head cartilage initially appears normal. Upon exposure to air, the cartilage affected by FAI turns reddish^29^. This region has significant clinical relevance as it corresponds to the area of osteophyte development in advanced osteoarthritis. However, till date, the origin of the reddening is not known. In contrast, healthy cartilage unaffected by FAI, remains whitish upon air contact.

Despite advancements in radiographic and biomedical analysis, elucidating the complex biochemical pathways of cartilage damaged as a result of FAI remains a challenge. To address these knowledge gaps, there is a critical need for analytical techniques that can provide a multi-faceted understanding of tissue composition and behavior. While LIBS, RS, and DRS offer individual strengths in probing elemental composition, molecular structure, and optical properties, respectively, their combined application offers a synergistic approach. The core motive of this study is to leverage the complementary nature of these label-free optical techniques to gain a deeper understanding of biochemical alterations in FAI-affected tissue, particularly focusing on the origin of the red staining of cartilage at the anterior femoral neck upon air contact. The novelty of this research is the first integration of LIBS, RS, and DRS on human FAI samples from the same study. The significance of this work lies in the newly proposed biochemical pathway model for FAI, providing crucial insights for future diagnostic and therapeutic interventions.

**Fig. 1:**
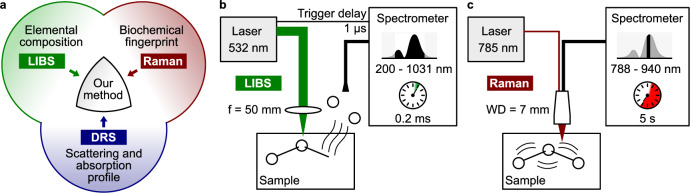
Schematic of the measurement set-ups. **a** Visualization of the combination of the different techniques. **b** LIBS set-up: A frequency-doubled, Nd:YAG laser is focused at the surface of the sample and ablates a few micrometers of material. The recombination from the generated plasma cloud is detected as LIBS signal. **c** RS set-up: The sample is positioned at the working distance of the probe, a diode laser is used for the excitation, and a spectrometer detects the back scattered light.

In this study, the capabilities in investigating FAI-affected tissue of three chosen technologies, namely RS and LIBS combined with DRS results from our previous study^29^ are discussed as shown in Fig. 1a. While LIBS and Raman have long been used in combination^30^, their application in medical and biochemical analysis remains limited, as noted in a 2021 review by ref. ^8^. Typical application include the nalysis of kidney stones (renal calculi) showing clear difference between different kinds of kidney stones^31^. In this study, results LIBS and RS combined with previous DRS results, which is summarized in Fig. 1.

## Results

### Laser-induced breakdown spectroscopy (LIBS)

**Fig. 2:**
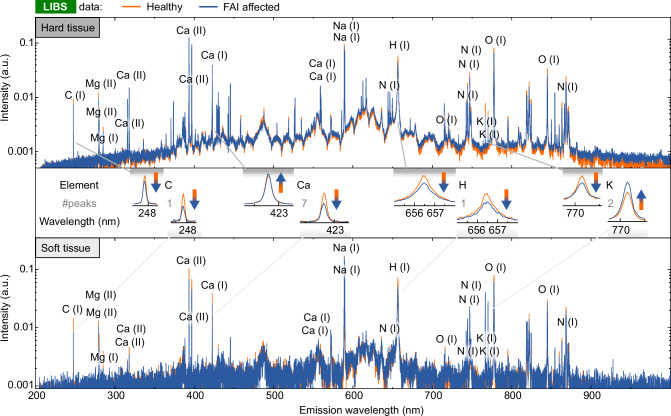
Averaged LIBS spectra of FAI affected and healthy reference tissue. Intensity values are scaled logarithmically. Air measurements have been discarded based on the measured nitrogen and Ca2+ peaks. All peaks used for further analysis are labelled. Top: The averaged spectrum from data sorted as hard tissue. Center: Example peaks for comparison. Bottom: The averaged spectrum from data sorted as soft tissue.

The results of the LIBS analysis for each individual peak are given in the the section attachment after the references (Tables 1 and 2). The mean spectra are shown in Fig. 2, where we separated the results for hard and soft tissue (see methods section for details). The majority of observable peaks are caused by calcium recombination. The peaks used for data analysis are labelled. In the LIBS spectra, calcium generates the majority of observable peaks Table 3.

**Table 1:** Detailed information about each analysed LIBS peak for the hard tissue

Element	Increased in FAI	*p*-value
C (I), 247.93 nm	False	5.0 ⋅ 10^−03^
Mg (II), 279.63 nm	False	1.1 ⋅ 10^−^^03^
Mg (II), 280.36 nm	False	2.3 ⋅ 10^−01^
Mg (I), 285.28 nm	True	4.7 ⋅ 10^−01^
Ca (II), 316.00 nm	True	7.8 ⋅ 10^−03^
Ca (II), 318.04 nm	True	1.1 ⋅ 10^−^^04^
Ca (II), 393.46 nm	True	3.7 ⋅ 10^−^^01^
Ca (II), 396.84 nm	True	2.4 ⋅ 10^−^^01^
Ca (I), 422.78 nm	True	9.5 ⋅ 10^−02^
Ca (I), 559.04 nm	True	1.5 ⋅ 10^−06^
Ca (I), 559.63 nm	True	2.4 ⋅ 10^−06^
Na (I), 589.15 nm	False	5.3 ⋅ 10^−^^08^
Na (I), 589.77 nm	False	1.5 ⋅ 10^−05^
N (I), 644.12 nm	True	7.2 ⋅ 10^−^^05^
H (I), 656.73 nm	False	7.9 ⋅ 10^−09^
O (I), 715.93 nm	True	4.3 ⋅ 10^−^^01^
N (I), 742.64 nm	False	6.8 ⋅ 10^−01^
N (I), 744.48 nm	False	1.6 ⋅ 10^−^^07^
N (I), 747.10 nm	False	1.3 ⋅ 10^−^^05^
K (I), 766.72 nm	False	3.6 ⋅ 10^−04^
K (I), 770.14 nm	False	1.5 ⋅ 10^−^^01^
O (I), 777.63 nm	False	1.6 ⋅ 10^−10^
O (I), 844.88 nm	False	1.4 ⋅ 10^−14^
N (I), 868.35 nm	False	8.8 ⋅ 10^−08^

**Table 2:** Detailed information about each analysed LIBS peak for the soft tissue

Element	Increased in FAI	*p*-value
C (I), 247.93 nm	False	1.3 ⋅ 10^−03^
Mg (II), 279.63 nm	True	8.2 ⋅ 10^−^^01^
Mg (II), 280.36 nm	False	7.3 ⋅ 10^−02^
Mg (I), 285.28 nm	False	9.6 ⋅ 10^−01^
Ca (II), 316.00 nm	False	9.9 ⋅ 10^−01^
Ca (II), 318.04 nm	False	9.5 ⋅ 10^−^^01^
Ca (II), 393.46 nm	False	2.3 ⋅ 10^−04^
Ca (II), 396.84 nm	False	1.9 ⋅ 10^−04^
Ca (I), 422.78 nm	False	3.0 ⋅ 10^−^^03^
Ca (I), 559.04 nm	False	6.9 ⋅ 10^−^^01^
Ca (I), 559.63 nm	False	6.5 ⋅ 10^−01^
Na (I), 589.15 nm	False	9.7 ⋅ 10^−^^01^
Na (I), 589.77 nm	True	4.8 ⋅ 10^−^^01^
N (I), 644.12 nm	False	6.4 ⋅ 10^−01^
H (I), 656.73 nm	False	3.5 ⋅ 10^−02^
O (I), 715.93 nm	False	8.8 ⋅ 10^−01^
N (I), 742.64 nm	True	3.5 ⋅ 10^−02^
N (I), 744.48 nm	False	8.2 ⋅ 10^−^^01^
N (I), 747.10 nm	True	4.7 ⋅ 10^−01^
K (I), 766.72 nm	True	1.5 ⋅ 10^−02^
K (I), 770.14 nm	True	7.3 ⋅ 10^−^^02^
O (I), 777.63 nm	False	1.6 ⋅ 10^−01^
O (I), 844.88 nm	True	7.5 ⋅ 10^−01^
N (I), 868.35 nm	False	3.3 ⋅ 10^−^^01^

**Table 3:** Information about the ANOVA for the FAI parameter

Component	df_*FAI*_	Sum sq. (FAI)	Mean sq (FAI)	F_*FAI*_	p_*FAI*_	$\partial {\eta }_{FAI}^{2}$
**Hydroxyproline**	2	27	14	34	1.5 ⋅ 10^−^^13^	0.25
C-C stretch	2	8.1	4.1	20	9.8 ⋅ 10^−^^9^	0.17
${{{{\boldsymbol{v}}}}}_{3}{{{{\boldsymbol{PO}}}}}_{4}^{3}$	2	230	120	190	2.1 ⋅ 10^−47^	0.65
**Phenylalanine**	2	2500	1300	130	1.1 ⋅ 10^−^^36^	0.56
**Pyranose ring**	2	150	76	130	4.1 ⋅ 10^−^^36^	0.55
${\nu }_{1}C{O}_{3}^{2-}$	2	39	20	17	1.6 ⋅ 10^−^^7^	0.14
**Pyranose ring**	2	43	22	120	9.5 ⋅ 10^−^^35^	0.54
**Amide III**	2	79	39	51	1.5 ⋅ 10^−18^	0.33
Lipid *CH*_2_ twist	2	1.6	0.78	7.4	0.00079	0.07
Tyrosine	2	0.24	0.12	1.9	0.15	0.02
**Amide III GAGs**	2	39	19	110	5.1 ⋅ 10^−33^	0.52
***CH***_2_ bend	2	96	48	108	8.1 ⋅ 10^−^^33^	0.52
Phenylring	2	0.49	0.25	5.4	0.0052	0.05
Tyrosine	2	40	3.0	27	3.4 ⋅ 10^−^^11^	0.04
Amide I	2	1.8	0.88	6.5	0.0019	0.06
**Carbonyl**	2	12	5.8	81	1.0 ⋅ 10^−^^26^	0.45

The bold components are seen as statistical relevant.

The hard tissue of FAI-affected patients presents a higher concentration of calcium than healthy individuals which is expected since increased levels of hydroxyapatite, the Ca-based main constituent of hard tissue, in FAI-affected samples is increased^32,33^. Both observations indicate elevated calcium levels. The reduction in carbon and hydrogen levels suggests decreased organic material content in FAI-affected tissue. The oxygen tends to be decreased in FAI-affected tissue despite its large presence in hydroxyapatite due to the reduced organic compounds in the FAI-affected tissue. Four out of five peaks demonstrate a decrease in nitrogen in the FAI-affected tissue, providing additional support the decrease in organic material. Potassium levels follow the same pattern as organic matter and, since it is largely found in cells, it can be regarded as a metric for organic matter as well.

The statistical significance for soft tissue analysis is limited due to fewer measurements. FAI-affected soft tissues contain less carbon, calcium, and hydrogen. A smaller amount of carbon indicates less organic tissue, while the decrease of calcium suggests that the mineralisation of the hard tissue collects the calcium from surrounding soft tissue as the calcium in hard tissue is used to grow bones. Hydrogen levels are significantly lower in FAI-affected tissue, which is consistent with the carbon peak and also indicates a reduction in organic tissue. All the peaks analyzed show a significant difference, while the calcium peaks exhibit low significance. All three peaks demonstrate consistent trends. Other differences between FAI and healthy tissue were negligible and are not discussed further.

### Raman spectroscopy (RS)

**Fig. 3:**
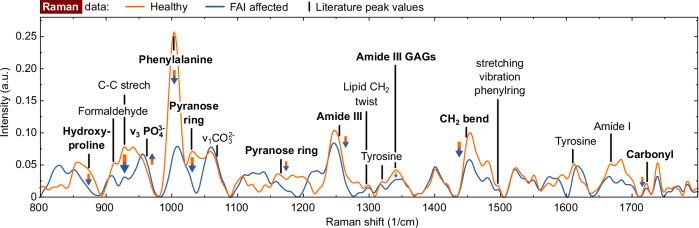
Mean Raman spectra of tissue affected by FAI and healthy reference tissue after denoising and baseline subtraction. All peaks for further analysis have been labelled, with the bold font indicating the peaks demonstrating significant differences (*p* < 0.005 and influence of peaks is larger than of the sample number).

Figure 3 depicts the averaged Raman spectra after denoising and baseline subtraction. Regardless of the statistical significance of each peak, there is an apparent decrease in almost all peaks that represent organic substances such as C-C stretch or pyranose ring in FAI affected tissues. Concurrently, the FAI affected tissue shows an increase in inorganic peaks (${\nu }_{3}P{O}_{4}^{3}$ and ${\nu }_{1}C{O}_{3}^{2-}$). These findings corroborate the LIBS data indicating reduced organic matter content. Furthermore previous studies have reported decreased cartilage cell density in FAI-affected tissue^34^.

The increased levels of ${v}_{3}P{O}_{4}^{3}$ and ${\nu }_{1}C{O}_{3}^{2-}$ in FAI affected tissue are caused by the increased concentraion of hydroxyapatite^35^. As an increase in the 1661 cm^−1^ peak of amide I indicates collagen quality change caused by ageing^16^, hydration/dehydration^36^, or radiological harm^37^, this observation demonstrates that the tissue affected by FAI is damaged. Nonetheless, it lacks statistical significance. Furthermore, there is a decline in the amount of hydroxyproline in FAI.

Additionally, the peak of phenylalanine is decreased in FAI-affected tissue. Phenylalanine is frequently transformed into tyrosine which, through 3,4-dihydroxyphenylalanine (DOPA), produces melanin via enzymatic oxidation. Nevertheless, the peaks related to tyrosine do not indicate any substantial differences. Further, both pyranose ring peaks are significantly reduced in FAI affected tissue, much more than other organic substances. The same is true for phenylalanine. Consequently, the presence of these two peaks infers that further loss is attributable to other biochemical reactions.

## Discussion and proposed FAI model

The FAI model is divided into two parts and primarily summarizes the findings from the LIBS and RS sections in conjunction with our prior DRS results^29^. The first part examines FAI-induced bone tissue alterations, demonstrating how the combination of DRS, Raman, and LIBS provides comprehensive insights when compared with previous studies. The second section summarises the factors not accounted for by the bone model and combines them with established biochemical pathways to explicate the red discoloration of FAI-affected tissue upon exposure to air.

**Fig. 4:**
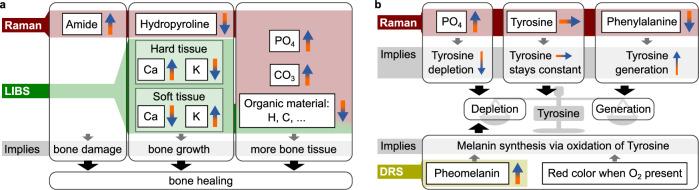
Summary of the findings and resulting bone models. **a** Bone growth model: Most of the findings are related to damage, growth and repair of the bone. **b** FAI model: Biochemical model for the reason of the red coloring of the FAI-affected tissue upon air contact.

Figure 4a, illustrates three distinct categories of alterations in FAI-affected tissue. Firstly, elevated levels of amide I reveal bone damage, which is an outcome of frequent impingement. Secondly, reduced hydroxypyroline for collagen stabilization can be observed. The hydroxylation of proline by prolyl hydroxylase or other electron-withdrawing reactions greatly enhances collagen’s conformational stability^38^. This indicates that the body responds to the damage by generating new bone. Moreover, there is an increase in calcium in hard tissue and a decrease in soft tissue, while the opposite holds true for potassium. Osteocytes predominantly store potassium intracellularly^39^. During bone strengthening, calcium accumulates from surrounding soft tissue. Fractures or microfractures initiate callus formation, activating blood cells and osteoblasts. Consequently, this leads to the reinforcement or repair of the bone affected by FAI. As a third observation, there is an increase in the amount of mineral components ($P{O}_{4}^{3}$ and $C{O}_{3}^{2-}$) in the tissue affected by FAI, which also shows an increase of bone tissue. This may be attributed to the association between FAI and deposition of calcium hydroxyapatite at the osteochondral junction shown by MRI techniques^32,33^. Additionally, LIBS indicates a lower concentration of organic tissue in general. Thus, there is more or hardened bone present. In summary, the bone damage, the body’s attempt to repair it, and the resulting increased levels of inorganic tissue are evidently shown by our measurements.

The previous model explains the majority of the findings. However, the bone model alone cannot account for the red discoloration upon exposure to air, the strong reduction of phenyalanaline, and the presence of pheomelanin in FAI affected tissue which could be shown by our previous DRS study^29^. To consider these points, the model has to be expanded, which is depicted in Fig. 4b. It is established that pheomelanin results from the enzymatic oxidation of Tyrosine catalysed by tyrosinase, through L-Dopa, Dopaquinone, and Cysteinyl DOPA^40,41^. First, tyrosinase catalyses the initial and rate-limiting step in the cascade of reactions that lead to melanin production from tyrosine^42^. More precisely, tyrosinase hydroxylates tyrosine to DOPA and catalyses the oxidation of DOPA to DOPAquinone^42^. Furthermore, the oxidation of Cysteinyl DOPA can occur under the appropriate conditions^43^. In addition to this reaction pathway, tyrosine is known to be essential for bone growth. However, under typical conditions in the body, the concentration of tyrosine required for melanin synthesis is approximately 20 times higher than that necessary for bone growth^40^. Hence, an increase in tyrosine would be expected in our measurements, which contradicts the constant measured amount of tyrosine. Therefore, there must be additional mechnisms contributing to a decrease in tyrosine and eventually balance the tyrosine level. When there is sudden contact with air, the concentration of oxygen rises, which subsequently promotes the reaction of tyrosine to pheomelanin, and the excess of tyrosine is then used to produce pheomelanin. As a result, the tyrosine present in the bone affected by FAI reacts suddenly and produces pheomelanin, causing an alteration in the tissue’s color to red.

The findings of this study are potentially of high clinical significance, as the mechanical concept of Cam-Type impingement suggests that the aspherical part of the femoral head is forced into the acetabulum during flexion and internal rotation, causing shear forces on the acetabular and femoral cartilage. In the diagnosis of FAI, radiographic examinations of the hip joint demonstrate obvious changes to the normal anatomy, in particular a reduced bony offset at the anterior femoral neck. To a limited extent, delayed gadolinium-enhanced magnetic resonance imaging (dGEMRIC MRI) allows biochemical analysis of FAI-affected tissue. These examinations are complex and have not become standard in everyday clinical practice. Thus, the assessment of the location and amount of bone resection in the treatment of FAI is based on the intra-operative macroscopic examination of the tissue and the mechanical testing of the head sphericity. In open surgery, the red coloring of the cartilage mentioned above provides additional valuable information about the correct resection site, but this discoloration is absent in the commonly performed arthroscopic treatment of FAI. Therefore, a tissue-sparing biochemical examination of the tissue would be of great clinical value. The results of this study provide a solid foundation for the development of intraoperative devices, not only for FAI-affected hips but also for all joints that may be affected by mechanical, degenerative, or traumatic cartilage injuries.

While the presented FAI model provides a potential biochemical pathway, some limitations of the study have to considered. First, the measurement of the different modalities was not performed on the same samples. Furthermore, for RS only three samples could be measured. Moreover, formaldehyde has an effect on the RS^44,45^, potentially altering the results. However, this problem is reduced as the healthy and FAI affected samples were both stored in formaldehyde solutions. Hence, it can be expected that both sample categories should see the same or at least similar alteration caused by the formaldehyde.

Despite these methodological limitations, the results of the study demonstrate the significant potential of the analytical techniques employed. The non-contact, all-optical analytical methods employed in this study demonstrate considerable utility in the biochemical assessment of FAI-affected tissue. Building upon our previous DRS analysis that identified pheomelanin in FAI-affected samples^29^, the integration of RS and LIBS has provided more comprehensive insights into the biochemical tissue composition. This multi-modal analysis-examining absorptive properties, scattering behavior, elemental composition, and molecular binding-enabled a more detailed characterization of the biochemical alterations associated with FAI progression.

A significant methodological advantage of this approach is that all measurements were obtained without markers, without direct contact with the sample, and without causing significant tissue damage. The combination of DRS, RS, and LIBS offers analytical capabilities that may be applicable to various biochemical investigations in orthopedic pathologies. The non-contact nature of these techniques makes them particularly suitable for potential in vivo applications, such as during arthroscopic procedures or laser surgery. Additionally, these technologies can operate at micrometer resolution, potentially enabling high spatial resolution analysis of affected tissues.

It should be noted that successful implementation of these analytical approaches requires interdisciplinary expertise, encompassing domain knowledge of biochemical pathways, technical understanding of optical spectroscopy, and advanced statistical analysis methods.

## Methods

### Statistics and reproducibility

Statistical analyses for LIBS and RS measurements were conducted independently due to the different sample sets and measurement techniques. For LIBS analysis, 21 biopsies from 18 patients were examined, with 5–10 randomly selected measurement points per sample and 25 individual measurements per spot. After filtering to remove out-of-focus measurements and classifying between hard and soft tissue, 965 spectra were obtained from white reference tissue (885 hard, 80 soft) and 1222 from FAI affected tissue (1153 hard, 69 soft). Statistical significance between FAI and healthy reference tissue was determined using the two-sided Mann–Whitney rank test with a significance level of 0.005 to adjust for multiple comparisons.

For RS, samples from three patients were analyzed, with 200 spectra initially recorded (100 from FAI tissue, 100 from healthy reference). After quality filtering based on formaldehyde peak presence, the total was reduced to 170 spectra (97 from FAI, 73 from healthy tissue). ANOVA was used to analyze the distribution of selected peaks, with significance threshold set at *p* < 0.0001. To account for the limited patient number, potential significance was only assumed if the partial eta-squared of FAI was at least similar to the partial eta-squared of inter-patient variation.

Data normalization was performed using L2 norm for LIBS data and formaldehyde peak normalization for Raman data. Reproducibility was ensured by multiple measurements at different sample locations, measurement point randomization to minimize experimental bias. Both intra- and inter-patient variations were considered in the Raman analysis to distinguish biological variation from disease-specific changes.

This is a summary of the statistical approaches; a more detailed description of each method, including specific processing steps and parameters, can be found in the respective method sections for LIBS and RS.

### Patients

LIBS analysis was conducted on 21 biopsies (one or two osteochondral samples from the anterior femoral head-neck junction) obtained from 18 patients during open surgery for treatment of Cam Type FAI at the Orthopaedic Clinic of the University of Bern, Switzerland. The patients were given full information about the study. The patients then gave their informed consent to participate in the study. The study was conducted in accordance with the tenets of the Declaration of Helsinki and was approved by the Cantonal Ethics Commission of Bern (KEK - decision of 15 October 2015).

After the procedure, the samples were stored in a 4% formaldehyde solution (Roti-Histofix 4%, Carl Roth). The air-induced color change enabled clear distinction between healthy and affected tissue areas. Therefore, histology was not performed. Each sample had the colored degeneration and an unaltered white border around it, which served as a healthy reference. From each of these samples, 5–10 measurement points were taken on the entire sample. LIBS measurements were obtained from both bone and cartilage tissue. An in-depth analysis of LIBS then allowed clear distinction between hard and soft tissue, based on molecular composition. This LIBS analysis was further supported by a Raman investigation of three additional samples. Figure 1b shows the measurement set-ups used in this study. The LIBS measurement was performed on different samples than the Raman measurements. For both modalities, measurement points were randomly selected across the sample to minimize experimental bias.

### Laser-induced breakdown spectroscopy (LIBS)

A frequency-doubled Nd:YAG laser (Q-smart 450, Quantel Laser, Les ulis cedex, France) with a repetition rate of 10 Hz, a pulse duration of 5 ns and a wavelength of 532 nm was used for the LIBS measurements. The mean pulse energy measured 80 mJ. A lens with a focal length of 50 mm was utilised to focus the beam just beneath the surface of the tissue sample. A 3D translation stage was also employed to relocate the measurement spot on the sample into the laser focus. A UV-enhanced 50 μm optical fiber was positioned above the sample to collect emission from the laser-induced plasma. The opposite end of the fibre was linked to a high-precision spectrograph (Mechelle ME 5000 Echelle, Andor, Belfast, UK). Equipped with an ICCD camera (A-DH334T-18F-03 USB iStar ICCD detector, Andor, Belfast, UK), the spectrometer has a spectral resolution (*λ*/*Δλ*) of 6000 within the 200–840 nm spectral range. The laser was directly connected to the spectrograph to trigger the detector measurements with each laser pulse.

All experiments were performed with a gate delay between the laser pulse and the detector of 1 μs and a gate width of 0.2 ms. In total, 21 samples from 18 patients were investigated with 6 to 10 spots per patient. Twenty-Five individual measurements were taken from each spot, with each measurement involving the ablation of a portion of the tissue. Thereby, first soft tissue and later hard tissue is measured by drilling into the tissue by laser ablation. Each ten LIBS pulses create a crater of approximately 300 μm in depth and diameter^5^. Due to the irregular shape of the sample, it is possible that the ablation was not within the tissue, resulting in plasma generation from the air. To remove such measurements, the nitrogen content is compared with the *Ca*^2+^ content. Nitrogen content is known to be very high in air, while *Ca*^2+^ content is low. The ratio of the peak intensity of *Ca*^2+^ at 392 nm to N at 822 nm^46^ was, therefore, used to filter out measurements in which the plasma had been generated in air. Spectra with ratios exceeding 0.5 (*n* = 412) were excluded from further analysis. The identified peaks were ultimately assigned to elements using information derived from the NIST Atomic Spectra Database^46^.

A comparable differentiation step was implemented in order to discern between hard and soft tissue areas. The tissue spectra vary significantly for both hard and soft tissue^6^, hence a distinct analysis was executed for these two tissue categories. The distinctive structure of the molecular matrix has a significant impact on the ablation process, resulting in differences in plasma quantity, temperature and hence, the intensity of the recorded peaks. Therefore, a comparable separation must also be conducted for LIBS data. The classification was based on the Ca (I) to K (I) ratio recorded at 616 nm and 766 nm^46^, which is known to exhibit the most significant variations between the cartilage and cortical bone^6^. Spectra with a ratio below 0.5 are classified as soft tissue (cartilage), while spectra with a ratio above 1.0 are classified as hard tissue (cortical bone)^6^. Intermediate spectra (*n* = 976) were excluded to ensure reliable classification.

As a result, 965 spectra were obtained from the white reference tissue, and 1222 from the FAI affected tissue. Among these spectra, 885 and 1153 were hard tissue for the white reference and FAI affected tissue, respectively. The remaining spectra comprised of 80 soft tissue spectra from the white reference tissue and 69 from the FAI affected tissue. Fewer soft tissue spectra were obtained because the thin cartilage layer was quickly penetrated by the laser pulses.

**Fig. 5:**
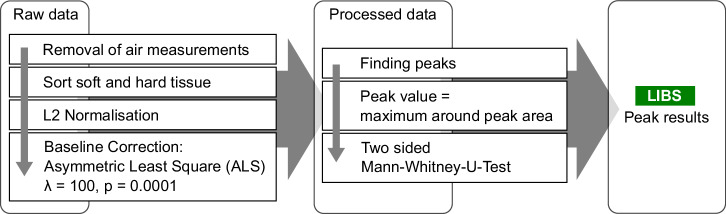
Schematic of the data processing algorithm used for the LIBS measurements. First, measurements that were out-of-focus are filtered out. The raw data is then normalized and sorted between soft and hard tissue based on the ratio of calcium and potassium. Afterwards, a base line correction is done, and the peak values are collected.

The technique for processing and analysing the LIBS data is outlined in Fig. 5. After removing artefacts and classifying into hard bone and cartilage, the spectra were normalised using L2 norm. The following step involved removing the offset via the asymmetric least square fit (ALS)^47^. The chosen weight was *p*_*als*_ = 0.0001, and a regularisation parameter of *λ*_*als*_ = 100 was employed. From the processed data, the peak values were subjected to the two-sided Mann–Whitney rank test for evaluation. As a large number of peaks were tested, a significance level of 0.005 was set to avoid significant results by chance.

### Raman spectroscopy (RS)

Due to the promising LIBS-results, Raman measurements were added. Only samples from three patients were available. The experimental setup is presented in Fig. 1c in a schematic manner. This setup is identical to the one we utilised in our previous study^29^ where we measured the bone and cartilage of FAI-affected tissue using DRS and RS. For RS, a diode laser (LASER-785-LAB-ADJ-S, Newport Corporation, Ocean Optics, Dunedin, USA) with a wavelength of 785 nm was used as the excitation source. The light is coupled into a fibre of a Raman coupled fibre probe (RIP-RPB-785-SMA-SMA, Ocean Optics, Dunedin, USA) via a SMA 905 coupling connector. The back-reflected light was captured and measured with a fibre-based spectrometer (QE65000 Spectrometer, Ocean Optics, Dunedin, USA). The Raman probe has an internal notchfilter for filtering out the excitation light.

**Fig. 6:**
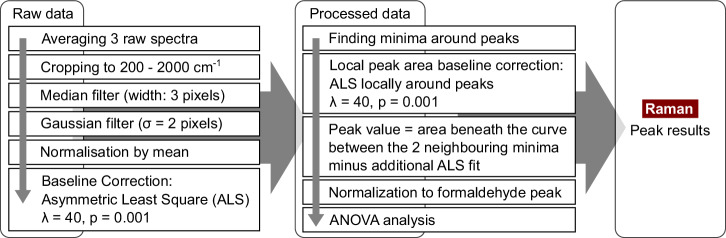
Schematic of the data processing algorithm used for the Raman measurements. The raw data is processed by median filtering to reduce the effect of artefacts. Afterwards, noise reduction is done by Gaussian smoothing. After a normalization step, ALS is used for the baseline subtraction of autofluorescence. Around the peaks, the minima of the spectra are found as marker for the peaks. For this area, a second ALS is done to access the real peak height. Finally, the peak area is taken, normalized to the formaldehyde peak, and an ANOVA is performed.

A total of 200 spectra were recorded, ranging from 788 to 940 nm or 52 to 2100 cm^−1^, with 100 obtained from the tissue affected by FAI and 100 from the healthy reference tissue. For each spot, five spectra were acquired with an integration time of 20 s each. As the Raman signal of tissue had displayed strong autofluorescence with an excitation wavelength of 785 nm, a correction procedure became necessary. Figure 6 details the implemented algorithm. Initially, the raw spectra of all three spots were averaged and then cropped to the spectral range of 200–2000 cm^−1^. A one-dimensional median filter, with a width of three pixels, was used to eliminate any artefacts from the sensor readout. The noise reduction process entailed utilising a two-pixel sigma Gaussian filter. Following this stage, the signal was normalised to the mean. The estimation of autofluorescence background was executed through ALS, with a *p*_*als*_ = 0.001 as its asymmetric weight and a regularisation parameter of *λ*_*als*_ = 40. Ultimately, the Raman signal was obtained by subtracting the autofluorescence signal.

From the processed signal, the maxima and minima were used to assign the Raman peaks. If a maximum was positioned more than 10 cm^−1^ away, the peak was discarded. For the localized peaks, an additional ALS was conducted using a weight of *p*_*als*_ = 0.0001 and a regularization parameter of *λ*_*als*_ = 0.1 to fit a function to the minima. The value of the peak was determined by calculating the area beneath the curve between the two neighbouring minima minus additional ALS fit.

Since RS is sensitive to formaldehyde, which can alter the Raman fingerprint slightly^44,45^, we normalized the Raman data using the formadlehyde peal at 911 cm^−1^ as the formalin peaks at 542, 1046, 1239, and 1492 cm^−1^ have considerable overlaps with other peaks. As the formaldehyde concentration was the same for all samples, these peaks are assumed to be independent of the sample under investigation. However, as the sample size varied, there may have been a slight difference in the final concentration, which can affect the Raman peaks of formaldehyde^48^. The Raman peaks may not always be sufficiently clear for data analysis. Consequently, such measurements are eliminated by verifying the existence of the formaldehyde peak. This filtering process reduced the total number of measurements to 170. Of these, 97 were from the FAI, and 73 from healthy tissue.

After normalisation, the distribution of selected peaks across all spectra was analysed. To achieve this, the statsmodel framework^49^ was used to perform an analysis of variance (ANOVA). The three parameters considered for the analysis were FAI, sample, and position on the sample. The latter two were selected to estimate intra- and inter-patient variation. As only three patients were measured, potential significance is only assumed if the partial eta-squared of the FAI ($\partial {\eta }_{\,{\mbox{FAI}}\,}^{2}$) was at least similar to the partial eta-squared of the inter-patient variation ($\partial {\eta }_{\,{\mbox{patients}}\,}^{2}$). Furthermore, as many peaks were examined, potential significance was only accepted if *p* < 0.0001.

### Reporting summary

Further information on research design is available in the Nature Portfolio Reporting Summary linked to this article.

## Supplementary information

reporting summary

## Data Availability

The data that support the findings of this study are available from the corresponding author upon reasonable request.

## Code availability

The code that was used to generate the findings of this study is available from the corresponding author upon reasonable request.
